# The great need to overcome osimertinib resistance in advanced non-small cell lung cancer: from combination strategies to fourth-generation tyrosine kinase inhibitors

**DOI:** 10.3389/fonc.2023.1308460

**Published:** 2024-01-09

**Authors:** Giuseppe Bronte, Alessia Belloni, Luana Calabrò, Lucio Crinò

**Affiliations:** ^1^ Clinic of Laboratory and Precision Medicine, National Institute of Health and Sciences on Ageing (IRCCS INRCA), Ancona, Italy; ^2^ Department of Clinical and Molecular Sciences (DISCLIMO), Università Politecnica delle Marche, Ancona, Italy; ^3^ Department of Oncology, University Hospital of Ferrara, Cona, Italy; ^4^ Department of Translational Medicine, University of Ferrara, Ferrara, Italy; ^5^ Department of Medical Oncology, IRCCS Istituto Romagnolo Per Lo Studio Dei Tumori (IRST) “Dino Amadori”, Meldola, Italy

**Keywords:** non-small cell lung cancer, osimertinib, resistance, tyrosine kinase inhibitor, allosteric inhibitor

## 1 Introduction

Lung cancer remains the leading cause of cancer-related deaths worldwide, surpassing the mortality of breast, colorectal and prostate cancers combined. The 5-year overall survival is around 18% including all stages of non-small cell lung cancer (NSCLC). It is likely that screening, targeted therapy, and immunotherapy will improve this poor prognosis in the coming years (1).

Currently, the choice of the treatment for patients with advanced NSCLC is based on the integrated evaluation of some parameters: histology (squamous versus non-squamous); presence of driver molecular alterations (sensitizing mutations of EGFR and/or BRAF, and rearrangements of ALK and/or ROS1 and/or NTRK); PD-L1 expression level; patient clinical characteristics such as age, performance status (PS) and comorbidities. The presence or absence of those driver molecular alterations allows distinguishing oncogene-addicted disease from non-oncogene-addicted disease, which presents different therapeutic approaches (2).

Many driver molecular alterations have been known in NSCLC till now. Some of these can be already targeted by means of specific drugs, while others could be targetable. These include: KRAS gene mutations (20-30%), EGFR (10-15% of Caucasian patients and up to 40% of Asian patients), BRAF (2-4%), ALK rearrangements (3-7%), ROS1 (1-2%), RET (1-2%), NTRK (0.5-1%), HER2 gene mutations (1-2%) and MET gene amplifications or mutations (2-4%) (3).

EGFR tyrosine kinase inhibitors (TKIs) represent the recommended first-line treatment in patients with advanced NSCLC and common mutations, i.e. exon 19 deletions (Ex19dels) and exon 21 point mutation (L858R). Many randomized phase III trials showed, in patients with advanced NSCLC and common EGFR mutations, the superiority of EGFR TKIs, i.e. gefitinib, erlotinib, afatinib, in the first line of treatment compared to standard platinum-based chemotherapy, in terms of both RR and PFS (4). The majority of patients treated with these drugs, around a half, develop the resistance point mutation T790M. To overcome this resistance mechanism, osimertinib was developed as second-line treatment. Subsequently its superiority in terms of overall survival (OS) over first- and second-generation TKIs was demonstrated also in the first-line setting (5, 6). As a consequence, osimertinib had been the best option for the treatment of advanced NSCLC patients with activating EGFR mutations until now (2). However, the recent results of FLAURA-2 trial and MARIPOSA trial showed that upfront combination strategies delayed resistance occurrence. Indeed, osimertinib plus platinum-pemetrexed and amivantamab plus lazertinib, respectively, achieved longer PFS compared to single agent osimertinib (7, 8).

## 2 Osimertinib resistance

Osimertinib has largely improved the survival of advanced NSCLC patients with EGFR mutation both in the first- or second-line setting, but the emergence of acquired resistance remains a great need.

The resistance mechanisms to osimertinib may be different depending on whether this drug is administered in the first or in the subsequent lines. In fact, osimertinib was initially developed exclusively for patients who had developed a T790M mutation during treatment with first or second generation TKIs. In this setting, the most frequent on-target resistance mechanism (approximately 14%) is the point mutation of EGFR exon 20, C797S. Instead, MET amplification occurred in 19% of patients, in 7% in conjunction with the C797S mutation. Furthermore, HER2 and PIK3CA amplifications, RET and NTRK rearrangements, and BRAF V600E mutation were each observed in 3–5% of cases (9, 10).

As regards the use of osimertinib as first-line treatment, some literature data reported an intrinsic resistance. The relative mechanisms include the HER2 and MET amplifications, as observed in *in vitro* studies, but also the combination of the KRAS G12D mutation and PTEN loss, and CDCP1 or AXL RNA overexpression, as reported in some NSCLC patients (11–13).

The main information about the acquired resistance to first-line osimertinib derives from the phase III trial FLAURA, while some other literature data are from case reports or small case series. Cell-free DNA from blood samples of patients included in FLAURA trial was analyzed via NGS (14). The authors did not find emergent T790M mutation. The most common resistance mechanism was MET amplification (15%), followed by less frequent EGFR amplification (9%), as well as C797S mutation (7%). S768I mutation or other combined EGFR mutations, such as exon 19 deletion + G724S (exon 18), L718Q (exon 18) + EGFR exon 20 insertion (exon 18 + 20), L718Q + C797S or L718Q + L797S (exon 18 + 20), are very rare (<1%). These findings suggest that secondary EGFR mutations are not the main mechanism of resistance to osimertinib.

Many EGFR-independent mechanisms were observed when resistance to first- or second-line osimertinib developed. These mechanisms are more evident with osimertinib than on-target mutations possibly because of its stronger EGFR inhibition compared to first or second-generation TKIs (15). Moreover, during the treatment with osimertinib EGFR-independent resistance mechanisms tend to occur earlier than EGFR-dependent ones, maybe because of pre-existent subclones rapidly arising under the selective pressure of treatment (16). This kind of mechanisms includes MET amplifications, HER2 amplifications, PI3KCA, BRAF and RAS mutations, ALK or RET rearrangements, cell cycle gene alterations (17). The ongoing ELIOS trial (NCT03239340) is designed to investigate prospectively the mechanisms of acquired resistance to first-line osimertinib, given that paired tissue biopsies (pre-treatment and at progression) are collected. Moreover, HER3 expression was found in around 80% of NSCLC and in more than 85% of those harboring EGFR activating mutations. It emerged as a further mechanism of resistance to EGFR TKIs and favors metastatic progression and worse prognosis (18).

Finally, phenotypic changes were also observed as resistance mechanisms in up to 15% of patients treated with first- or later-line osimertinib. These include the epithelial-mesenchymal transition and the transformation of adenocarcinoma to small-cell carcinoma, this latter associated with RB1 and TP53 mutations (19).

Patients experiencing progression during first-line osimertinib should receive chemotherapy regimens used in non-squamous NSCLC. The combination of carboplatin, paclitaxel, bevacizumab and atezolizumab could represent a treatment option for them (20). Anti-PD-1/PD-L1 drugs as monotherapy have not proven to be a successful option in this subgroup of patients (21). However, three phase III trials studied chemoimmunotherapy after osimertinib resistance. CheckMate-722 confirmed the lack of benefit in these patients (22). KEYNOTE-789 and ORIENT-31 are still ongoing.

To prolong the benefit obtainable with osimertinib, similarly to first-generation TKIs, this drug could be continued “beyond progression” in selected cases with some conditions, such as exclusively radiological progression of mild entity, slow tumor kinetics, absence of clinical symptoms, and in case of oligoprogressive disease local therapies can be associated (23).

Here, we discuss the evolution of treatment strategies to achieve the best outcomes for patients progressing during first-line osimertinib. The majority of the studies addressing this aim are still ongoing.

## 3 Combination strategies

Many trials were designed to combine osimertinib with other new targeted agents directed against the emerging resistance mechanisms. These drugs can be classified in specific inhibitors, antibody-drug conjugates, and bispecific antibodies (24) (**Table 1**).

**Table 1:** Drugs in clinical development for patients progressing during or after osimertinib.

Target	Category	Name	3^rd^ gen EGFR TKI combined	Trial phase advancement
EGFR-directed	Allosteric inhibitors	BLU-701	Yes	I/II
		BLU-945	Yes	I/II
		BBT-176	No	I/II
		JIN-A02	No	I/II
Other-target-directed	MET inhibitors	Savolitinib	Yes	III
		Tepotinib	Yes	II
		Capmatinib	Yes	III
	MEK inhibitors	Selumetinib	Yes	Ib
	ERK inhibitors	ERAS-007	Yes	I/II
	PI3K inhibitors	TQ-B3525	Yes	I/II
	mTOR inhibitors	Sapanisertib	Yes	I
	CDK4/6 inhibitors	Abemaciclib	Yes	II
	AXL inhibitors	DS-1205c	Yes	I
	JAK1 inhibitors	Itacitinib	Yes	I/II
	AURKA inhibitors	Alisertib	Yes	I
	EGFR-MET bispecific antibodies	Amivantamab	Yes	III
	Antibody-drug conjugate	Trastuzumab deruxtecan	No	Ib
		Patritumab deruxtecan	Yes	III
		Datopotomab deruxtecan	No	III
		SKB264	Yes	III
		Telisotuzumab vedotin	Yes	III

Given that MET alterations are the most frequent resistance mechanism to osimertinib, three MET inhibitors were developed and combined with osimertinib, i.e. savolitinib, tepotinib and capmatinib. However, other specific inhibitors are available against MEK (selumetinib), AXL (DS-1205c), ERK (ERAS-007), mTOR (sapanisertib), PI3Kα/δ (TQ-B3525), JAK1 (itacitinib), CDK4/6 (abemaciclib), AURKA (alisertib).

Antibody-drug conjugates (ADC) are composed of a link between a specific monoclonal antibody directed against a tumor molecule and a cytotoxic payload. After the bond of the monoclonal antibody with its own target, the drug is internalized and the linker is degenerated releasing the cytotoxic payload, with consequent antitumor effect. Some of these drugs have been already available in trials for NSCLC patients and specifically target MET (telisotuzumab vedotin), HER2 (trastuzumab deruxtecan), HER3 (patritumab deruxtecan), TROP2 (datopotamab deruxtecan and SKB264). All these antibody-drug conjugates are studied after osimertinib resistance. However, only patritumab deruxtecan and telisotuzumab vedotin are combined with osimertinib. Interestingly, the efficacy results from phase I and phase II trials with patritumab deruxtecan were achieved across a broad range of pretreatment HER3 expression in the tumor membrane and across the various EGFR-TKI resistance mechanisms (25, 26).

Finally, the category of bispecific antibodies in this setting is represented only by amivantamab until now. This is a fully humanized antibody directed against mutated EGFR and mutated or amplified MET. It exerts its function both via the inhibition of the ligand binding and antibody-dependent cellular cytotoxicity (27). This drug is combined with lazertinib, a potent brain-penetrant third-generation EGFR TKI, and investigated in three clinical trials (CHRYSALIS, CHRYSALIS-2, MARIPOSA-2) for patients progressing during or after osimertinib.

ORCHARD is a biomarker-directed Phase II platform trial. This study is evaluating the optimal treatment strategy depending on the underlying resistance mechanism to first-line osimertinib. For this reason, treatment assignment is based on a molecular tumor characterization from a tissue biopsy performed at progression during osimertinib. Each patient will be assigned to treatment with the combination of osimertinib and a specific targeted drug for the resistance mechanism detected, e.g. osimertinib + savolitinib for MET amplification, osimertinib + necitumumab for EGFR amplification, and so on. If a resistance mechanism is not found, the patient will be assigned to platinum-based chemotherapy with durvalumab. Those patients with resistance mechanisms not targetable will be treated according to local practice. This trial also includes an adaptive design to allow the addition of new emerging drugs (28). Recently, an interim analysis of safety and efficacy showed that osimertinib + necitumumab in patients with a secondary gene alteration in EGFR, i.e. amplification, L718 or G724 mutation, exon 20 insertion, met futility criterion so that recruitment for this treatment was closed (29).

## 4 Fourth-generation EGFR-TKIs

The therapeutic strategies for the acquired resistance to osimertinib we discussed before are suitable for EGFR-independent resistance mechanisms, which are more frequent for patients treated with first-line osimertinib. However, some of these patients develop on-target EGFR mutations, such as C797S point mutation in exon 20, mainly in *cis* with the activating mutation. To target this alteration a specific drug does not yet exist. Brigatinib, an ALK inhibitor showed activity against cells with this mutation, but it needed the combination with cetuximab to be effective *in vivo* (30). In a retrospective analysis including 15 patients who developed resistance to osimertinib because of C797S mutation, brigatinib plus cetuximab achieved the 10% objective response rate and 60% disease control rate (31).

Among the various EGFR inhibitors under development, there are new TKIs, which are “fourth generation” or allosteric EGFR inhibitors (**Table 1**). It means that these drugs bind to the receptor outside the ATP pocket of the kinase domain. This bond selectively alters EGFR conformation and bypasses the resistance mechanisms mediated by new mutations in the ATP-binding domain, mainly C797S (32). Among these drugs, a limited activity was obtained with EAI-045, the first one identified in this class, when used as a single agent, but it has to be combined with cetuximab to be effective in cell lines with triple EGFR mutations (L858R/T790M/C797S). Similarly, JBJ-04-125-02 achieved increased cell death and more effective inhibition of cell proliferation when combined with osimertinib, compared with single agent alone (33). CH7233163, a further fourth generation EGFR TKI, exhibited even more potent antitumor activity against the EGFR triple mutation (ex19del/T790M/C797S) (34). Some other allosteric EGFR inhibitors were included in the design of phase I/II clinical trials, i.e. BLU-701 (HARMONY trial), BLU-945 (SYMPHONY trial), BBT-176, JIN-A02. The first two of these are studied both as single agent and in combination with osimertinib in patients who progressed during or after a previous EGFR TKI.

## 5 Discussion

**Figure 1:**
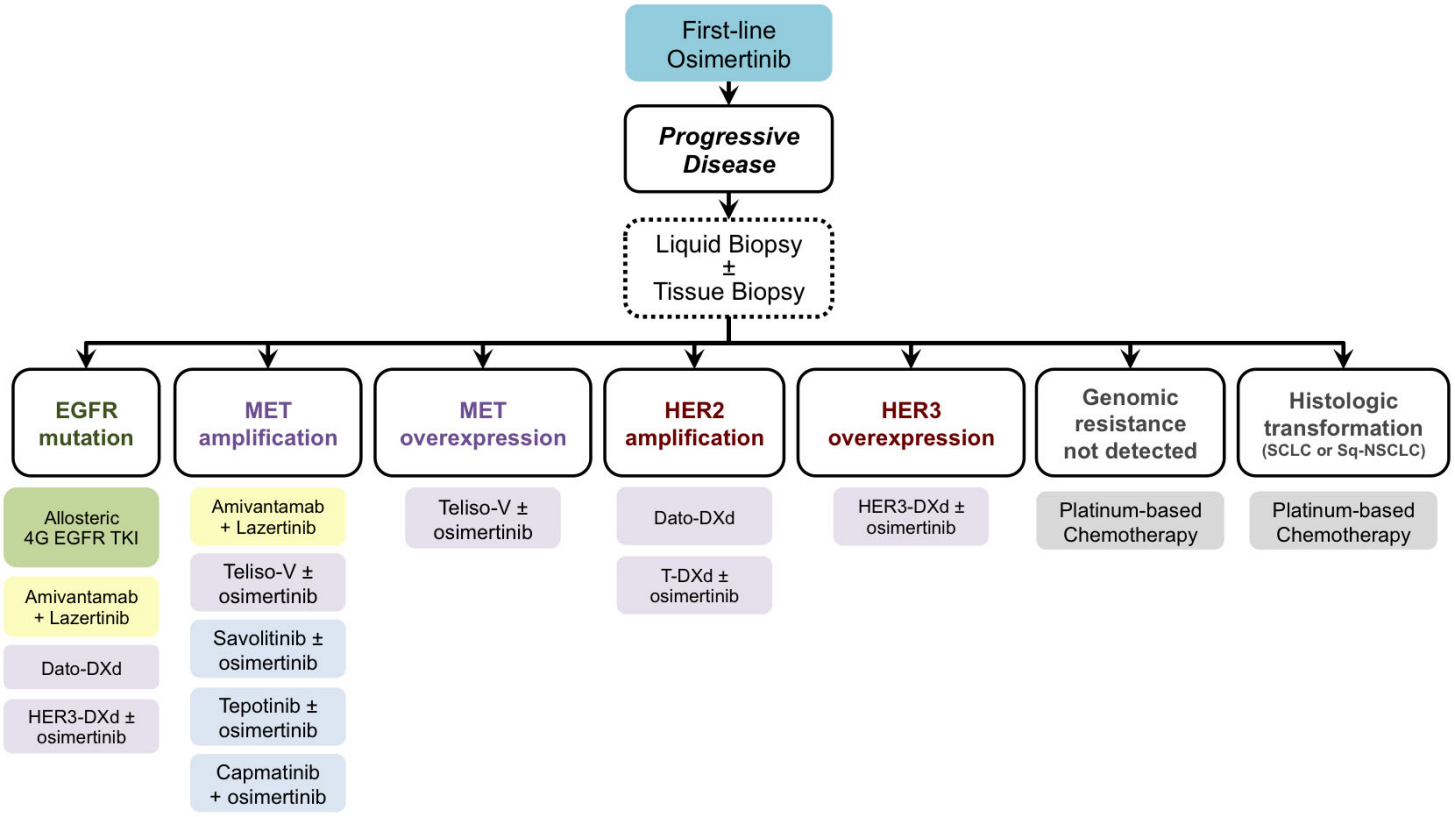
Future scenario for treating patients who developed resistance during or after osimertinib according to post-progression molecular characterization. EGFR, Epidermal Growth Factor Receptor; MET, MET Proto-Oncogene, Receptor Tyrosine Kinase; HER2, Human Epidermal Growth Factor Receptor 2; HER3, Human Epidermal Growth Factor Receptor 3; 4G, fourth generation; Dato-DXd, Datopotamab Deruxtecan; HER3-DXd, Patritumab Deruxtecan; Teliso-V, Telisotuzumab Vedotin; T-DXd, Trastuzumab Deruxtecan; SCLC, small cell lung cancer; Sq-NSCLC, squamous non-small cell lung cancer.

From the scenario of these new therapeutic strategies emerges the possibility of avoiding or delaying the chemotherapy option for patients who develop resistance to first-line osimertinib. We still do not have sufficient efficacy data available to allow considering some therapeutic strategies over others. However, based on the molecular targets at which these new drugs are directed, when patients treated with osimertinib become refractory to this drug, it is necessary to evaluate which resistance mechanism has developed. The search for these resistance mechanisms involves the need to carry out tissue biopsies, possibly associated with a blood sample for the detection of gene alterations in cell-free DNA. This combined, liquid and tissue, evaluation would allow defining the prevailing resistance mechanism, on the basis of which the most appropriate molecular treatment can be indicated. In principle, the therapeutic choice could be oriented towards combination strategies of osimertinib plus a specific inhibitor or antibody-drug conjugate or bispecific antibody in the case of EGFR-independent resistance mechanisms (e.g. MET overexpression or MET amplification, as highlighted in the biomarker analysis of CHRYSALIS-2 trial) (35). Instead, the use of allosteric EGFR TKIs would be more suitable in case of the prevalence of new EGFR mutations, especially at the ATP-binding site. A relevant issue that could be resolved by the results of these ongoing clinical trials concerns the usefulness of combining these new drugs with the continuation of osimertinib versus its suspension. We summarized a possible future scenario in **Figure 1** to attribute a next-generation therapeutic option to each of the more frequent mechanisms of resistance to first-line osimertinib. On the basis of the findings that are emerging from many clinical trials, we suppose that in the next future oncologists will be able to address each patient, who experience resistance to upfront osimertinib, toward a different treatment strategy according to the molecular alterations highlighted via circulating cell-free DNA and/or DNA from tumor tissue biopsy. However, the results of upfront combination strategies, such as those investigated in FLAURA-2 and MARIPOSA trial, will further change the scenario, and we think that a different spectrum of resistance mechanisms could emerge. To face with the complexity of gene alterations that can lead to the resistance to first-line treatment, i.e. osimertinib single agent or combination strategies, liquid biopsy will be mandatory and, if not informative, it can be completed with tissue biopsy. However, we believe that the design of platform trials could be the best option to manage the complexity of resistance occurrence.

## Author contributions

GB: Conceptualization, Data curation, Formal Analysis, Methodology, Writing – original draft, Writing – review & editing. AB: Data curation, Investigation, Software, Visualization, Writing – original draft, Writing – review & editing. LCa: Data curation, Formal Analysis, Investigation, Visualization, Writing – original draft, Writing – review & editing. LCr: Conceptualization, Data curation, Investigation, Supervision, Writing – original draft, Writing – review & editing.

## Glossary

**Table:**

ADC	Antibody-drug conjugate
ALK	anaplastic lymphoma kinase
ATP	Adenosine Triphosphate
AXL	AXL receptor tyrosine kinase
BRAF	v-raf murine sarcoma viral oncogene homolog B1
CDCP1	CUB-domain-containing protein 1
CDK4/6	Cyclin-dependent kinase 4 and 6
EGFR	Epidermal Growth Factor Receptor
ERK	Extracellular Signal-Regulated Kinase
HER2	Human Epidermal Growth Factor Receptor 2
HER3	Human Epidermal Growth Factor Receptor 3
JAK1	Janus kinase 1
KRAS	Kristen Rat Sarcoma viral oncogene
MEK	Mitogen-activated protein kinase kinase
MET	MET Proto-Oncogene, Receptor Tyrosine Kinase
mTOR	Mammalian target of rapamycin
NGS	next-generation sequencing
NSCLC	Non-Small Cell Lung Cancer
NTRK	Neurotrophic tyrosine receptor kinase
OS	Overall survival
PD-1	Programmed death-1
PD-L1	programmed death-ligand 1
PFS	Progression-free survival
PI3Kα/δ	Phosphoinositide 3-kinases α/δ
PI3KCA	Phosphatidylinositol-4,5-bisphosphate 3-kinase catalytic subunit alpha
PTEN	Phosphatase and TENsin homolog deleted on chromosome 10
RB1	retinoblastoma susceptibility gene 1
RET	rearranged during transfection
ROS1	ROS Proto-Oncogene 1
RR	Response rate
TKI	Tyrosine-Kinase Inhibitor
TP53	Tumor protein P53
TROP2	trophoblast antigen 2
